# Orbital Engineering in Sillén–Aurivillius Phase Bismuth Oxyiodide Photocatalysts through Interlayer Interactions

**DOI:** 10.1021/acs.chemmater.3c00932

**Published:** 2023-07-12

**Authors:** Kanta Ogawa, Hajime Suzuki, Aron Walsh, Ryu Abe

**Affiliations:** † Centre for Processable Electronics and Department of Materials, Imperial College London, Exhibition Road, London SW7 2AZ, U.K.; ‡ Department of Energy and Hydrocarbon Chemistry, Graduate School of Engineering, Kyoto University, Nishikyo-ku, Kyoto 615-8510, Japan

## Abstract

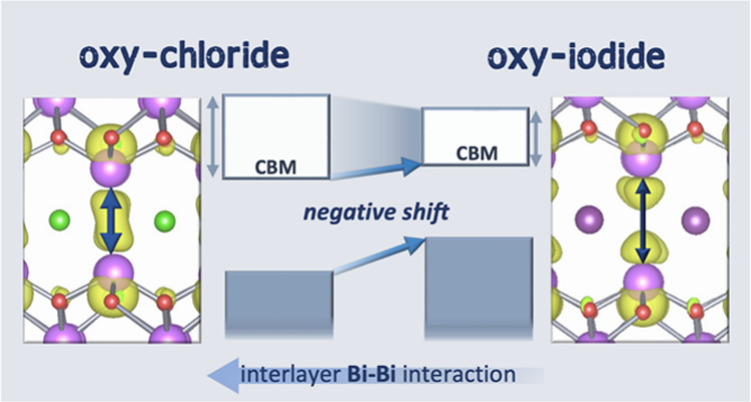

Multicomponent inorganic compounds containing post-transition-metal cations such as Sn, Pb, and Bi are a promising class of photocatalysts, but their structure–property relationships remain difficult to decipher. Here, we report three novel bismuth-based layered oxyiodides, the Sillén–Aurivillius phase Bi_4_NbO_8_I, Bi_5_BaTi_3_O_14_I, and Bi_6_NbWO_14_I. We show that the interlayer Bi–Bi interaction is a key to controlling the electronic structure. The replacement of the halide layer from Cl to I negatively shifts not only the valence band but also the conduction band, thus providing lower electron affinity without sacrificing photoabsorption. The suppressed interlayer chemical interaction between the 6p orbitals of the Bi lone-pair cations reduces the conduction bandwidth. These oxyiodides have narrower band gaps and show much higher water oxidation activities under visible light than their chloride counterparts. The design strategy has not only provided three novel Bi-based photocatalysts for water splitting but also offers a pathway to control the optoelectronic properties of a wider class of lone-pair (ns^2^np^0^) semiconductors.

## Introduction

Semiconductors containing post-transition metals are promising candidates for efficient solar-to-energy conversion owing to the electronic structure contributions of the s^2^ lone-pair electrons, e.g., as found for Bi(III) or Pb(II).^1−3^ These cation s^2^ orbitals hybridize with the anion p orbitals to form the valence band maximum (VBM) that determines the oxidation potential of the compound. For example, Bi 6s–O 2p hybridization reduces O 2p localization and results in a valence band more negative than conventional oxides such as TiO_2_ or SrTiO_3_, which enhances the visible-light photocatalytic response (e.g., in BiVO_4_,^4,5^ Bi_4_NbO_8_Cl,^2,6^ and Pb_2_Ti_2_O_5.4_F_1.2_^7^). Similarly, Pb 6s–O 2p hybridization in PbWO_4_ not only shifts the VBM negatively but also enhances the hole mobility.^8^ In the related lead halide perovskites, lone-pair hybridization is also key to their defect-tolerant nature.^9^ In contrast to the valence band, the conduction band minimum (CBM) is largely formed by the empty and more diffuse cation p orbitals, which affords good electron conductivity owing to the low effective mass and spatially delocalized conduction band.^10^

In the field of photocatalysis, Bi-based materials have emerged as promising photocatalysts for dye degradation,^11,12^ water splitting,^6,13,14^ and CO_2_ reduction.^15^ An important family is Bi (or Pb)-based oxyhalides with layered crystal structures. The common moiety in their structures is the Bi/Pb oxide-based fluorite-like layer, [M_2_O_2_], intergrown with single, double, or triple halide layers, [X], [X_2_], or [M′*_x_*X_3_], which is the so-called Sillén phase. Sillén phase materials include BiOCl, PbBiO_2_Cl, and PbBi_3_O_4_Cl_3_ photocatalysts.^16,17^ The Sillén phases can further intergrow with an Aurivillius phase containing a perovskite layer, of general formula [Bi_2_O_2_][A_*n*–1_B_*n*_O_3*n*+1_], producing a Sillén–Aurivillius phase of the general formula [A_2_O_2_][X_*n*_][A_2_O_2_][A′_*m*–1_B_*m*_O_3*m*+1_], represented by its simplest member Bi_4_NbO_8_Cl (i.e., *n* = 1, *m* = 1), which itself is a promising water-splitting photocatalyst.^18^ These Sillén and Sillén–Aurivillius photocatalysts also feature electronic signatures of the lone-pair cations, including an elevated valence band suitable for visible-light water splitting, along with robustness against self-oxidation by holes. Notably, the latter has been challenging to achieve for low-electronegative anions such as S^2–^ or N^3–^ due to facile oxidation by photogenerated holes. Additionally, the lower part of the conduction band of Sillén and Sillén–Aurivillius mainly consists of cation p orbitals favorable for electron transfer,^1,10,19^ while in conventional photocatalysts, more localized cation d (Ti 3d, V 3d, Nb 4d, Ta 5d, W 5d) orbitals are present.^20^

Owing to the specific electronic structure of Sillén and Sillén–Aurivillius compounds due to the s^2^ lone-pair electrons of Bi or Pb, a unique strategy is required to control and design the material properties such as band gaps, band edge positions, and carrier mobilities. While the cation–anion orbital interactions described earlier are one component, there are other factors, such as the coordination environment (i.e., the Madelung site potential), which contribute to the unique VBM character.^21^ In addition, it has been shown that the introduction of less ionic iodine in Bi_3_Ba_2_Nb_2_O_11_X with a double perovskite layer (*n* = 2) electrostatically destabilizes oxygen sites in the perovskite layer and modifies the valence band energy.^22^ On the other hand, design strategies for the conduction band have not yet been established. Although the electronic structure of Bi-based layered oxyhalides has been calculated based on density functional theory (DFT),^2,3,6,14,23,24^ few studies probe the details of the conduction band. While the negative shifting of the CBM of the Bi-based layered oxychloride has been achieved by changing the intralayer interaction of Bi and O in a Sillén Bi_2_MO_4_Cl (*M* = Bi, La, Y), this shift is accompanied by breaking the Bi–O bonds, decreasing photoconductivity, and widening the band bap, which significantly deteriorates the photocatalytic activity.^25^

**Figure 1:**
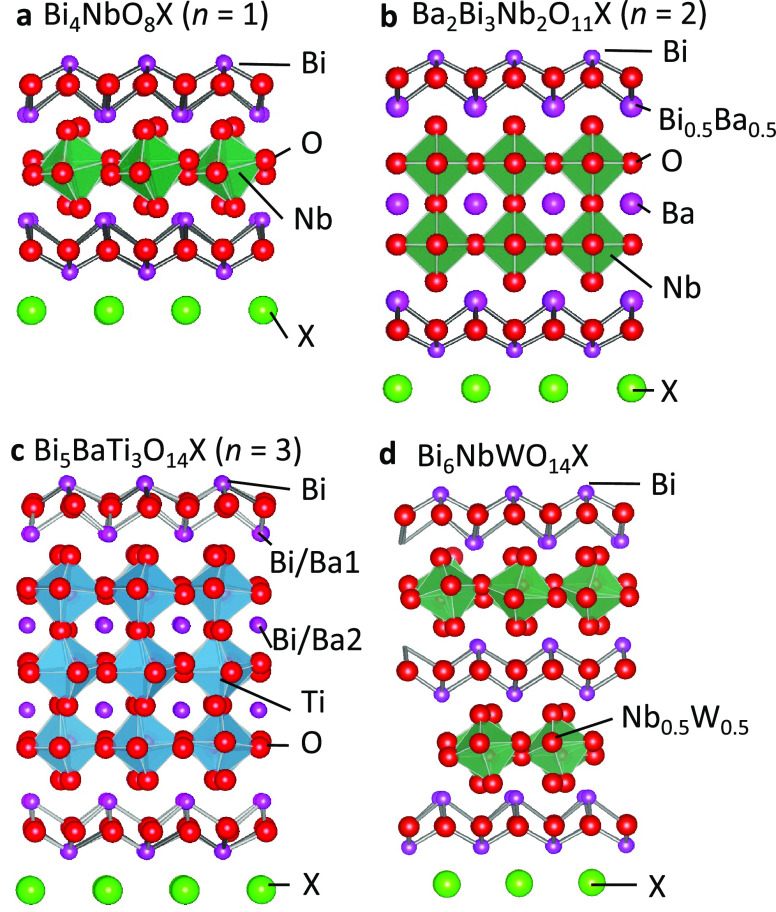
Crystal Structures of Sillén–Aurivillius oxyhalides (a) Bi_4_NbO_8_X (*n* = 1), (b) Bi_3_Ba_2_Nb_2_O_11_X (*n* = 2), and (c) Bi_5_BaTi_3_O_14_X (*n* = 3) and (d) Sillén–Aurivillius-related oxyhalide Bi_6_NbWO_14_X. For *n* = 3, the occupancy ratios in Bi/Ba1 and Bi/Ba2 sites are 0.63:0.37 and 0.87:0.13, respectively.

In this study, we demonstrate that the conduction band can be controlled without widening the band gap or sacrificing photocatalytic activity. This is achieved by changing the interlayer cation–cation interactions. Although Sillén–Aurivillius oxyhalides exhibit great structural variety,^18^ most synthesis reports are on the chlorides with few on oxyiodides^26,27^ because the oxyiodides had been considered to be unstable as photocatalysts until the discovery of the first oxyiodide water oxidation photocatalyst Bi_3_Ba_2_Nb_2_O_11_I.^22^ Here, we synthesized three novel layered perovskite oxyiodides, Bi_4_NbO_8_I, Bi_5_BaTi_3_O_14_I, and Bi_6_NbWO_14_I (Figure 1a–c), by introducing iodine into various Sillén–Aurivillius oxyhalides beyond *n* = 2. The iodine negatively shifts both band edges, which is in stark contrast to the case of *n* = 2. The origin of the conduction band shift is discussed from the perspective of the interlayer Bi–Bi interaction. The developed oxyiodides have narrower band gaps than the oxychlorides owing to the elevated valence bands, functioning as water oxidation photocatalysts under visible light with much higher activities than their chloride counterparts.

## Results and Discussion

### Synthesis

**Figure 2:**
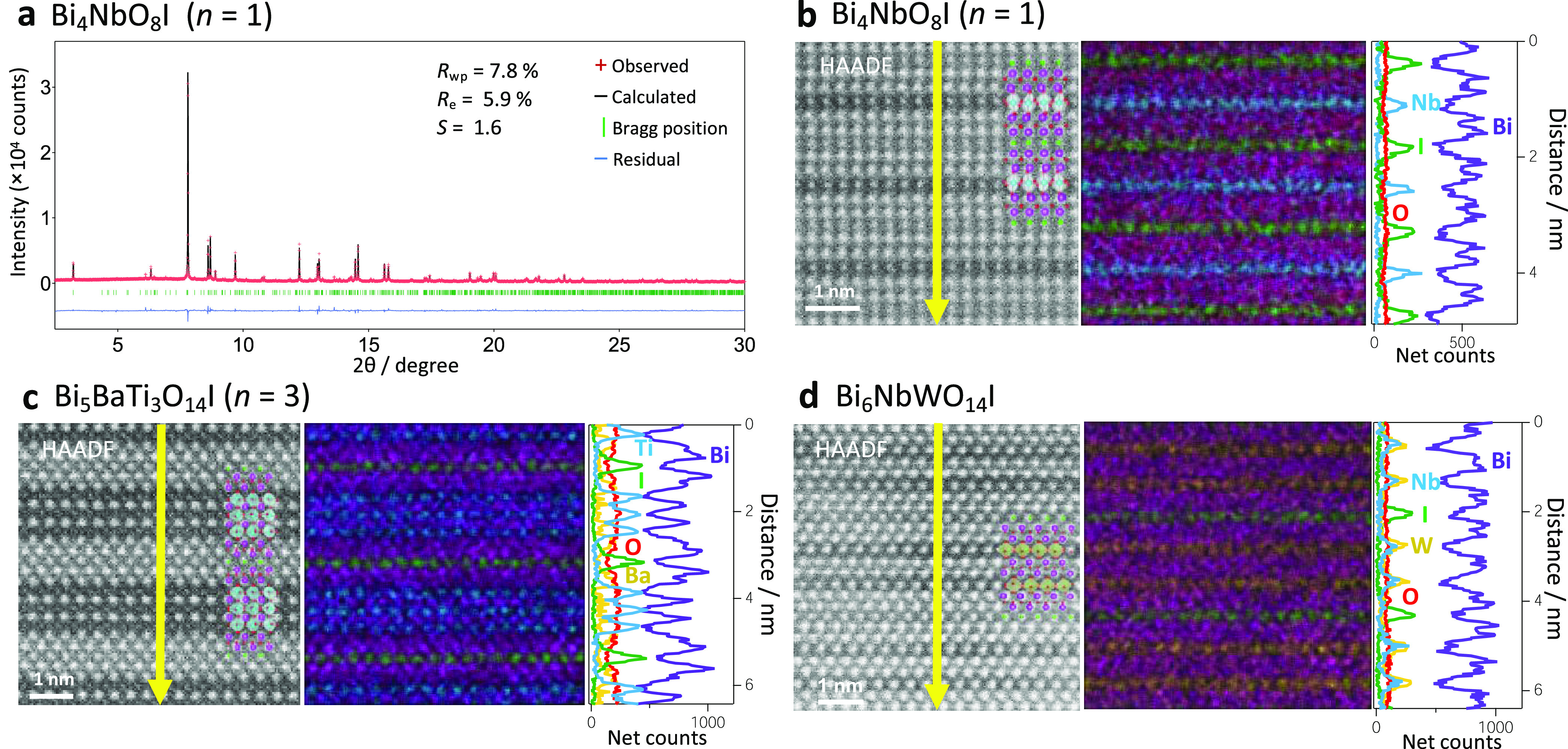
(a) Rietveld refinement of the SXRD pattern of prepared Bi_4_NbO_8_I. The refined structure is shown in Table S1. (b–d) HAADF-STEM images: atomic-resolution STEM-EDX elemental maps of (b) Bi_4_NbO_8_I along the [100]*_t_* direction, (c) Bi_5_BaTi_3_O_14_I along the [110]*_t_* direction, and (d) Bi_6_NbWO_14_I along the [110]*_t_* direction.

The three oxyiodides were synthesized via the solid-state reaction method. Figure 2a shows the synchrotron powder X-ray diffraction (SXRD) pattern of Bi_4_NbO_8_I, where Rietveld refinement confirmed the successful synthesis of the targeted material. The structure was further analyzed using neutron powder diffraction (NPD; Figure S1) to produce the refined structure in Table S1. Notably, Bi_4_NbO_8_I has been considered difficult to be synthesized because of the lattice mismatch between the perovskite [NbO_4_] layer and the fluorite [Bi_2_O_2_] layer.^26^ We employed Bi_3_NbO_7_, which has an oxygen-deficient fluorite structure,^28^ as a precursor of Nb species (Figure S2), while the previous attempt used a “rigid” oxide containing perovskite structure of the NbO_4_ unit, such as BiNbO_4_.^26^ This result indicates that not only the lattice matching but also the choice of the precursor materials affects the success or failure of synthesis of the targeted layered structures. We also successfully synthesized Bi_5_BaTi_3_O_14_I (*n* = 3) and Bi_6_NbWO_14_I (Figures S3 and S4, XRD patterns). High-angle annular dark-field scanning transmission electron microscopy (HAADF-STEM) images recorded parallel to the layers, along with the STEM/energy-dispersive X-ray spectroscopy (EDX) line scan analysis and elemental mapping (Figures 2b–d and S5–S7), are consistent with each crystal structure obtained from the XRD analysis. SEM-EDX mapping images of these oxyhalides show a uniform distribution of the constituted elements at a stoichiometric elemental ratio, further confirming the successful formation of these products (Figure S8).

### Band Gaps and Band Edge Positions

**Figure 3:**
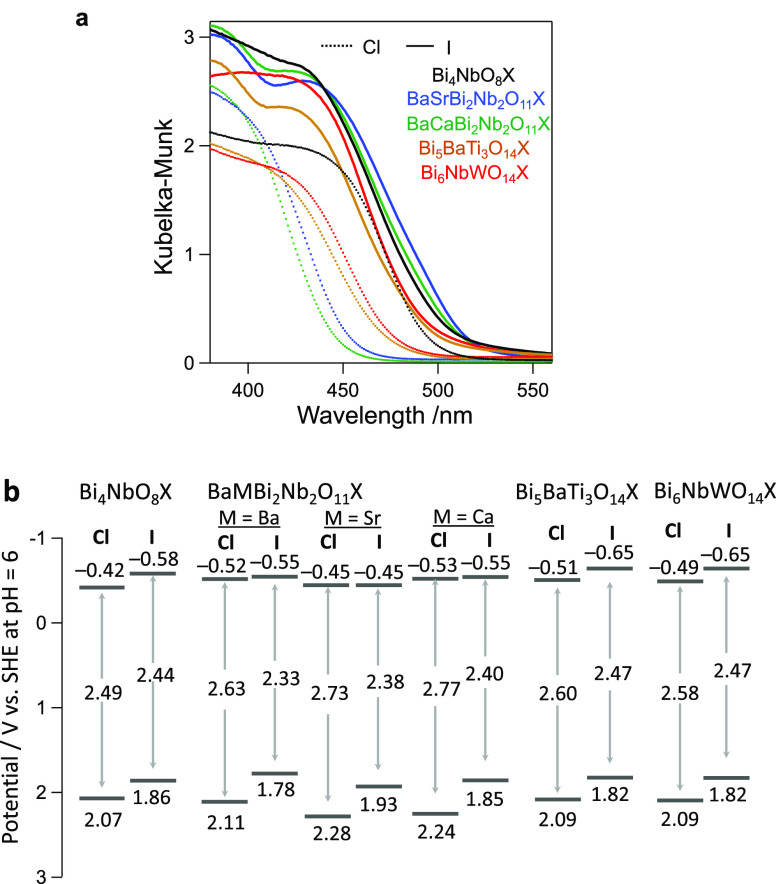
Optical absorption spectra (a) and band edge positions (b) of the series of oxychlorides and oxyiodides: Bi_4_NbO_8_X, BaMBi_3_Nb_2_O_11_X (M = Sr, Ca), Bi_5_BaTi_3_O_14_X, and Bi_6_NbWO_14_X. The band edge positions were estimated from Mott–Schottky plots in 0.1 M phosphate buffer solution (pH 6), as shown in Figure S9. Considering the n-type characters of these materials, the flat-band potentials were considered the conduction band minima.

The oxyiodides have narrower band gaps than the corresponding chloride counterparts because of the negatively shifted valence bands, as with the previously reported case of Ba_2_Bi_3_Nb_2_O_11_I (*n* = 2)^22^ (Figure 3). Figure 3b summarizes the band edge positions of the oxyiodides and the corresponding oxychloride. Based on the n-type character of these materials (Figure S9), the flat-band potential determined via Mott–Schottky analysis was considered the bottom of the conduction band.^29^ In addition to the three novel oxyiodides, the band positions of BaMBi_3_Nb_2_O_11_I (M = Ba, Sr, Ca) with the double perovskite layer (*n* = 2; Figure S4) were also shown. While the *n* = 2 with M = Ba was reported as a photocatalyst,^22^ the optical properties of its Sr and Ca substitutes have remained elusive despite there being a synthesis report.^27^ The valence band energies of all of the oxyiodides are negative compared to those of the chloride counterparts, being mainly composed of O 2p (Figure S10). The high polarizability of iodine energetically destabilizes the oxygen in the perovskite layer, contributing to the negatively shifted VBM^22^ (Figure S11).

A notable difference from the case of *n* = 2 is the conduction band position shifted by iodine introduction. While the iodine introduction to *n* = 2 exert little influence on the CBM,^22^ the other oxyiodides (Bi_4_NbO_8_I, Bi_5_BaTi_3_O_14_I, and Bi_6_NbWO_14_I) have about 0.14–0.16 eV negatively shifted compared to its chloride counterparts (Figure 3b). Namely, both band edges are negatively shifted in energy by iodine introduction, where the degree of the shift is larger for the valence band, enabling a negative conduction band shift without sacrificing photoabsorption.

### Origin of the Conduction Band Shift

**Figure 4:**
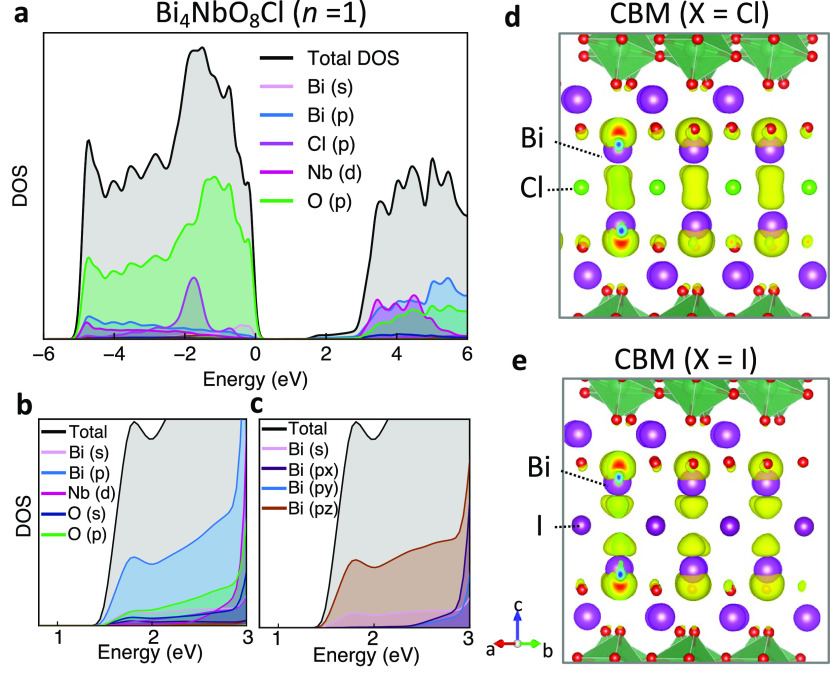
(a–c) Projected electronic density of states (PDOS) of Bi_4_NbO_8_Cl calculated by DFT. (d, e) Charge distribution of the lower part of the conduction band minimum (CBM) for Bi_4_NbO_8_X, where X = (d) Cl and (e) I.

To gain deeper insights into the origins of the observed behavior, we performed quantum chemical analysis using DFT. Figure 4a shows the calculated electronic density of states of Bi_4_NbO_8_Cl. While the upper valence band is composed of O 2p orbitals, the lower conduction band shows highly dispersed (low density) nature mainly from Bi 6p*_z_* (Figure 4b,c). As shown in Figure 4d, the lower conduction band is characterized by charge density connecting two Bi atoms across the halide layer; it is formed by the overlap of Bi 6p*_z_* orbitals. This observation is consistent with previous reports on Bi-based layered oxyhalides,^23,30^ although its origin has not been addressed. Here, we propose an explanation by considering the revised lone-pair model^1^ and formation via interlayer band formation.

**Figure 5:**
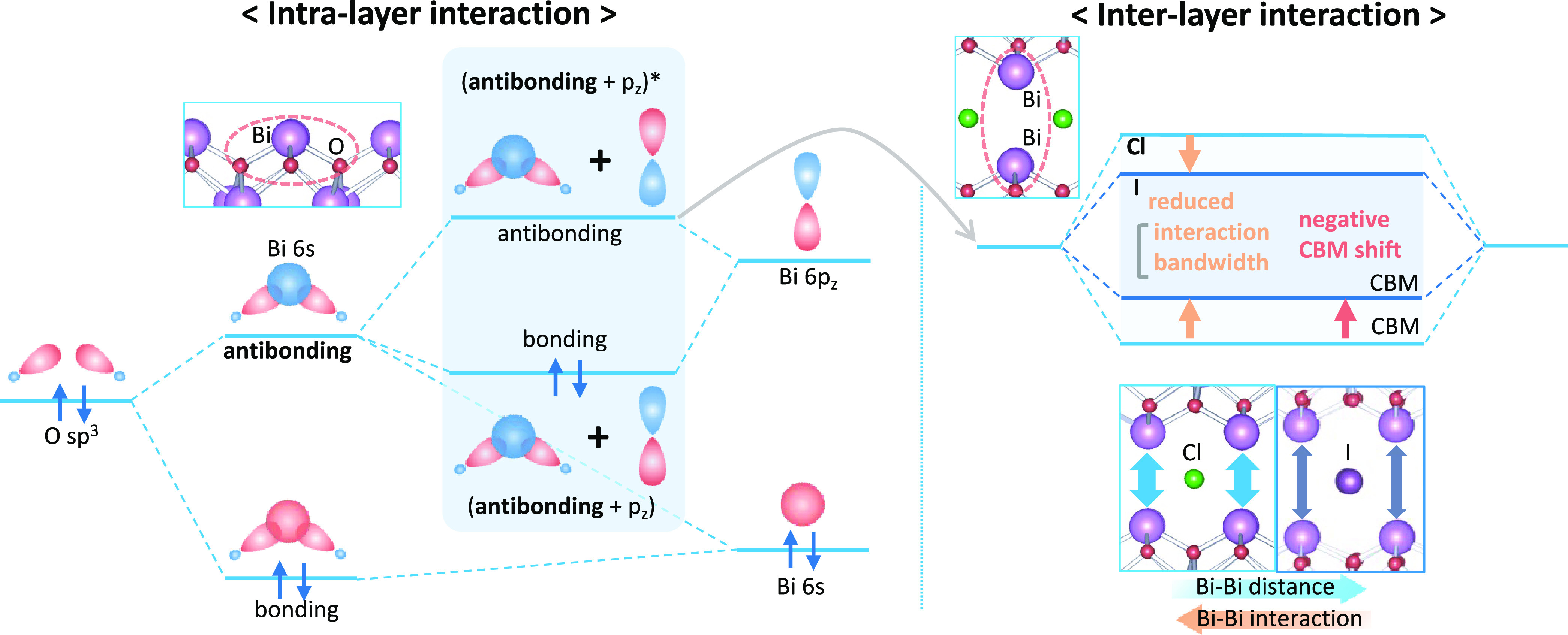
Formation of the conduction band of the Sillén–Aurivillius compounds via the intra- and interlayer interactions. Through the intralayer interaction (left side), the antibonding Bi 6s/O 2p combination further interacts with Bi 6p*_z_* to produce occupied bonding (antibonding + p*_z_*) and unoccupied antibonding (antibonding + p*_z_*)* levels. The latter further interacts with another (antibonding + p*_z_*)* via the interlayer interaction to form the conduction band. The resulting conduction band minimum (right side) is affected by the interlayer Bi–Bi distance, which can be altered by the halide layer. On the other hand, the occupied (antibonding + p*_z_*) level lies within the valence band, while the perovskite layer contributes to the bottom of the valence band (Figure S13).

Let us start with the intralayer interaction between Bi and O in the O–Bi–O block in the fluorite layer. The active orbitals of the ionic building blocks are Bi 6s^2^6p^0^ and O 2p^6^. Overlap between Bi 6s and O 2p results in filled bonding and antibonding combinations. The antibonding combination is formed in the upper valence band (Figure 5). However, the secondary mixing of the nominally empty Bi 6p orbitals results in energetic stabilization with a stereochemically active Bi lone pair. Most studies have focused on the valence band effects (e.g., BiVO_4_,^4,5^ Bi_4_NbO_8_Cl,^2,6^ and Pb_2_Ti_2_O_5.4_F_1.2_^7^) that arise from the filled bonding component of Bi 6p. Here, we are concerned with the empty antibonding component that forms the lower conduction band.

In Bi_4_NbO_8_Cl and other Sillén and Sillén–Aurivillius phases, two Bi atoms in different fluorite layers face each other across the halide layer. Umezawa et al. have shown that the CBM of SnO with a layered structure is formed by the interlayer Sn–Sn interaction.^31^ The interlayer Bi–Bi distance in the present materials (e.g., 3.9 Å in Bi_4_NbO_8_Cl)^32,33^ is comparable to the Sn–Sn distance in SnO (3.8 Å),^31^ which suggests that the interlayer interaction should also be considered in the present case. Such interlayer interaction can form a band (Figure 5). This description can explain the characteristic charge density shown in Figure 4d, where the conduction band is derived from the Bi–Bi 6p interaction. Crystal orbital Hamilton population analysis further confirms the favorable chemical interaction between adjacent Bi sites (Figure S12).

The negative shift by replacing Cl with I can be explained using this model. The Bi–Bi interlayer interaction is lowered by iodine introduction because replacement of Cl with I having a larger ionic size increases the Bi–Bi distance (Figure 4e). This change narrows the conduction bandwidth, which results in a negative shift of the CBM (Figure 5). Note that the change in the average Bi–O distance of the Bi next to the halide layer accompanied by the replacement of Cl with I is small (from 2.22 to 2.23 Å).

**Figure 6:**
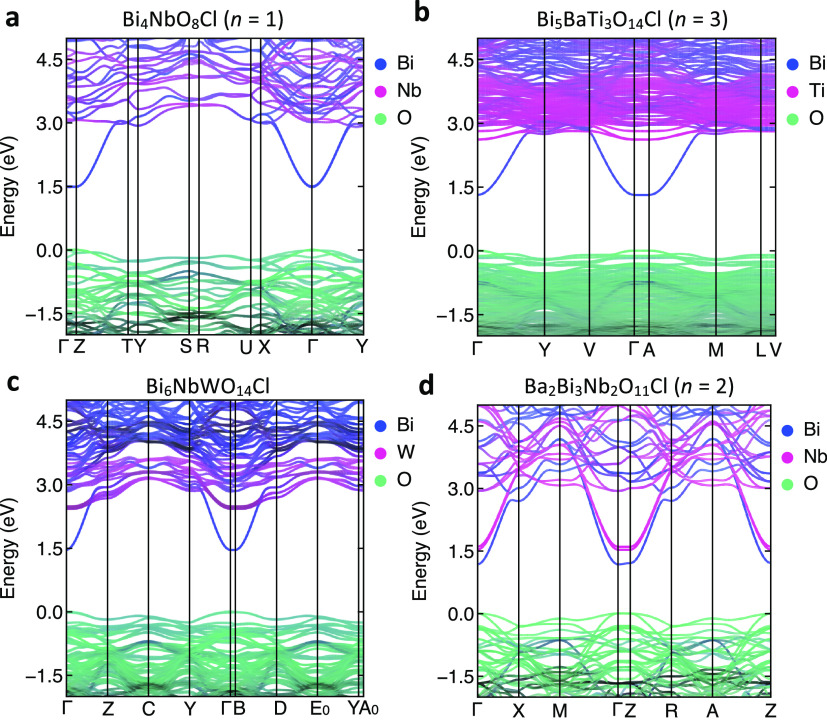
Electronic band structures of (a) Bi_4_NbO_8_Cl (*n* = 1), (b) Bi_5_BaTi_3_O_14_Cl (*n* = 3), (c) Bi_6_NbWO_14_Cl, and (d) Ba_2_Bi_3_Nb_2_O_11_Cl (*n* = 2).

In contrast to three novel oxyiodides (Bi_4_NbO_8_I (*n* = 1), Bi_5_BaTi_3_O_14_I (*n* = 3), and Bi_6_NbWO_14_I), the iodine introduction to BaMBi_3_Nb_2_O_11_Cl (*n* = 2) exerts little influence on the conduction band position (Figure 3b), although it increases the Bi–Bi interlayer distance. The notable difference is found in the density of states (Figure S14); while Bi 6p forms the CBM of Bi_4_NbO_8_Cl (*n* = 1), Bi_5_BaTi_3_O_14_Cl (*n* = 3), and Bi_6_NbWO_14_Cl, the B-site cation in the perovskite layer (Nb 4d) also contributes to the lower conduction band in Ba_2_Bi_3_Nb_2_O_11_Cl (*n* = 2). This difference is clearly seen in band dispersion (Figure 6), where the conduction bands of Bi_4_NbO_8_Cl, Bi_5_BaTi_3_O_14_Cl, and Bi_6_NbWO_14_Cl are derived from highly dispersive Bi 6p, while for *n* = 2, Nb 4d forms a second dispersive band slightly higher in energy. For the iodide of *n* = 2, this Nb d*_x_*_2–*y*2_ band actually forms the CBM; the increased Bi–Bi distance reduces the Bi-derived bandwidth (Figure S15b), but it no longer alters the Nb-derived CBM position. We note that the highly dispersive nature of the Nb band only in *n* = 2 may be due to the less tilted octahedron in the perovskite layer (Figure 1). Nb d_*x*2–*y*2_, which is the lowest energy orbital in the compressed octahedral geometry of the perovskite layer of *n* = 2 (Figure S15d), forms the lowest energy bond via π* interaction with O 2p (Figure S15e). Smaller tilting usually provides stronger π* interaction (i.e., higher dispersion of d orbital).^34^ In fact, the introduction of distortion to the perovskite layer in *n* = 2 significantly reduces the Nb contribution to the bottom of the conduction band (Figure S16). The more compressed Nb octahedra in the iodide of *n* = 2 (Figure S15f) may also contribute to the small shift of the CBM by further stabilizing the Nb 4d_*x*2–*y*2_ orbital.

In the present Sillén–Aurivillius materials, while the lower conduction band is largely composed of the fluorite layer, the upper valence band is composed of the perovskite layer.^14,22,35^ Their positions are dominated by different factors; the CBM is affected by the interlayer Bi–Bi distance of the two fluorite layers, while the VBM is affected by the energetic stability of the oxygen in the perovskite layer. The iodine introduction exerts influence on each band edge through different mechanisms (i.e., the elongated Bi–Bi distance and the electrostatic destabilization of the O site, respectively), which enables the negative shift of both band positions simultaneously. Thus, the CBM negative shift occurs without sacrificing photoabsorption. This is in stark contrast to SnO, where the same factor determines the positions of both the valence and conduction bands.^31^ Therein, decreasing the interlayer Sn–Sn interaction narrows the bandwidth of both bands, resulting in the negative shift of CBM, the positive shift of the VBM, and thus a widened band gap.

### Photocatalytic Activity

Table 1 summarizes the photocatalytic water oxidation activities of each sample under visible light in the presence of electron acceptors (Ag^+^). All of the oxyiodides function as water oxidation photocatalysts and show O_2_ evolution. This is in contrast to the case of Bi_2_MO_4_Cl, where the negative shift of the conduction band is accompanied by the loss of photocatalytic activity due to the broken Bi–O bonds. The photocatalytic activity of each oxyiodide is found to be superior to that of the oxychloride counterpart.

**Table 1:** Photocatalytic Water Oxidation Activities of Each Sample under Visible Light in the Presence of Ag^+^ Electron Acceptors^a^

		initial rate of O_2_ evolution (μmol h^–1^)	
no.	compound	Cl	I
1	Bi_4_NbO_8_X (*n* = 1)	4.8	41.5
2	Ba_2_Bi_3_Nb_2_O_11_X (*n* = 2)	4.7	37.6
3	BaSrBi_3_Nb_2_O_11_X	7.7	62.0
4	BaCaBi_3_Nb_2_O_11_X	6.2	32.3
5	Bi_5_BaTi_3_O_14_X (*n* = 3)	4.3	24.2
6	Bi_6_NbWO_14_X	13.8	89.6

a Reaction conditions: photocatalyst (0.1 g) dispersed in aqueous AgNO_3_ solution (8 mM, 100 mL); light source, Xe lamp (300 W) fitted with an L42 cutoff filter for visible-light irradiation (λ > 400 nm).

Considering that the iodides show higher photocatalytic activity even under monochromatic light (i.e., have higher apparent quantum efficiency at 405 nm) than the chloride counterparts (Table S2), the positive influence of iodine should extend beyond the band gap narrowing. Indeed, the carrier effective masses are affected (Table S3). Notably, iodine introduction decreases the calculated hole effective mass for every material. Iodine introduction decreases the electron effective mass for Bi_4_NbO_8_X (*n* = 1), Bi_5_BaTi_3_O_14_X (*n* = 3), and Bi_6_NbWO_14_X but increased that of Ba_2_Bi_3_Nb_2_O_11_X (*n* = 2). This is probably due to the change of the lower part of the conduction band from Bi 6p to localized Nb 4d in *n* = 2, which can relate to the H_2_ evolution activity from a sacrificial hole scavenger (Figure S17). By calculating the dielectric constants (Table S4), we further computed the carrier mobilities within the Fröhlich polaron model, motivated by the polar bonding nature of these materials similar to lead halide perovskites^36^ and antimony chalcogenides.^37^ The iodides show higher electron mobilities except for *n* = 2 (Table 2). On the other hand, the hole mobilities of the iodide are much higher than those of the chlorides for every structure. In the photocatalytic activity shown in Table 1, Ag^+^ was employed as a sacrificial electron acceptor and the water oxidation reaction can be a rate-determining step. Therefore, the increased hole mobilities can be a contributing factor to the increased photocatalytic activity by iodine introduction.

**Table 2:** Calculated Average Mobilities (cm^2^ V^–1^ s^–1^) along the Layer (*x*, *y* Plane) of Electrons (e) and Holes (h) at 300 K within the Fröhlich Polaron Model

	Cl		I	
	e	h	e	h
Bi_4_NbO_8_X (*n* = 1)	110.3	1.3	148.2	19.6
Ba_2_Bi_3_Nb_2_O_11_X (*n* = 2)	80.3	1.1	50.1	8.3
Bi_5_BaTi_3_O_14_X (*n* = 3)	125.6	1.2	154.4	1.8
Bi_6_NbWO_14_X	90.1	0.4	174.4	1.7

**Figure 7:**
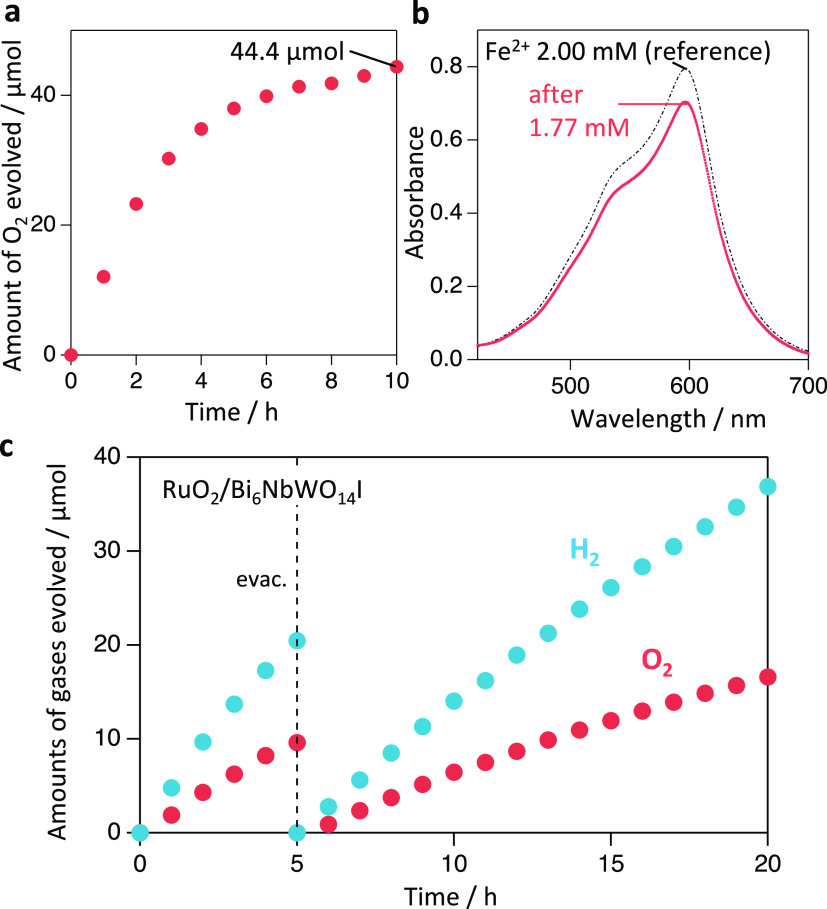
(a) O_2_ evolution over the RuO_2_-loaded Bi_6_NbWO_14_I sample in an aqueous Fe(NO_3_)_3_ solution (8 mM, 100 mL) at pH 2.4 under visible-light irradiation (λ > 400 nm). (b) Optical absorption spectra of Fe^2+^ after the photocatalytic reaction of Figure 6a detected as the Fe^2+^-2,4,6-tris(2-pyridyl)-1,3,5-triazine (TPTZ) complex. Fe^2+^ (1.77 mM, 177 μmol) is equivalent to 44.3 μmol O_2_. (c) H_2_ and O_2_ evolution over a mixture of RuO_2_-loaded Bi_6_NbWO_14_I and Ru/SrTiO_3_:Rh (50 mg each) in FeCl_3_ aqueous solution (2 mM, 100 mL) at pH 2.4 under visible-light irradiation (λ > 400 nm).

We used Bi_6_NbWO_14_I, showing the highest photocatalytic activity as an O_2_ evolution photocatalyst, in Z-scheme water splitting. RuO_2_-loaded Bi_6_NbWO_14_I achieved O_2_ evolution in the presence of Fe^3+^, which is the oxidant of the Fe^3+^/Fe^2+^ redox mediator, under visible-light illumination (Figure 7a). The amount of Fe^2+^ converted from Fe^3+^ by the photoexcited electrons in this half-reaction was determined by complexometric determination (Figure 7b), which was exactly four times the O_2_ evolved, confirming the stoichiometric redox reaction (i.e., 4Fe^3+^ + 2H_2_O → O_2_ + 4Fe^2+^ + 4H^+^). The gradual decrease of the activity in Figure 7a can be caused by the decreased concentration of Fe^2+^ and/or the increased rate of reverse reaction including Fe^2+^ (i.e., Fe^2+^ + h^+^ → Fe^3+^). Subsequently, visible-light water splitting using RuO_2_-loaded Bi_6_NbWO_14_I was conducted with the Fe^3+^/Fe^2+^ couple as the redox mediator and Ru-loaded strontium titanate doped with Rh cations^38^ (Ru/SrTiO_3_:Rh) as the H_2_-evolution photocatalyst. As shown in Figure 7c, H_2_ and O_2_ evolve stoichiometrically at steady rates, demonstrating Z-scheme water splitting under visible light.

## Conclusions

In this study, three new members of the Sillén–Aurivillius oxyiodide family were successfully synthesized. While iodine introduction to the Sillén–Aurivillius compound with *n* = 2 exerts little influence on the position of the CBM, the CBMs of Bi_4_NbO_8_X (*n* = 1), Bi_5_BaTi_3_O_14_X (*n* = 3), and Bi_6_NbWO_14_X negatively shift with iodine introduction, where the Bi–Bi interlayer interaction plays an important role. These Sillén–Aurivillius oxyiodides show photocatalytic O_2_ evolution activity much higher than their oxychloride counterparts. The present study demonstrated the negative shift of the conduction band by introducing iodine without sacrificing the photoabsorptivity and photocatalytic activity. Further modification of the charge of each layer and making solid solutions of Cl and I will control the Bi–Bi interlayer interaction, enabling fine tuning of the band positions for targeted reactions. The present strategy not only develops the three novel Bi-based photocatalysts for water splitting but also offer a new insight into controlling the optoelectronic properties and band positions of the ns^2^np^0^ semiconductors.

## Experimental Section

### Synthesis

All of the oxyhalides were prepared by solid-state reactions. In the case of Bi_3_Ba_2_Nb_2_O_11_X (X = Cl, Br, I), Sillén-type BaBiO_2_X and Aurivillius-type Bi_3_BaNb_2_O_9_ were mixed in the 1.05:1 composition and heated in air (X = Cl, Br) in an evacuated silica tube (X = I, and Cl for a certain case) at 800 °C for 20 h.^27^ BaBiO_2_X precursors were prepared by calcining a stoichiometric mixture of BaCO_3_ (FUJIFILM Wako Pure Chemical Corp.) and BiOX in air (X = Cl, Br) in an evacuated silica tube (X = I) at 800 °C for 20 h. BiOCl was purchased from FUJIFILM Wako Pure Chemical Corp., while BiOBr and BiOI were synthesized by a soft liquid deposition method;^39^ 5 mmol Bi(NO_3_)_3_·5H_2_O (FUJIFILM Wako Pure Chemical Corp.) was dispersed in 30 mL of ethanol and mixed with the solution of 5 mmol KX (X = Br or I) (FUJIFILM Wako Pure Chemical Corp.) dissolved in 10 mL of pure water. After 5 h of stirring at room temperature, the precipitate was collected by centrifugation, washed several times with water and ethanol, and finally dried in air at 60 °C. Bi_3_BaNb_2_O_9_ was prepared by calcinating the mixture of Bi_2_O_3_ (FUJIFILM Wako Pure Chemical Corp.), BaCO_3_, and Nb_2_O_5_ (FUJIFILM Wako Pure Chemical Corp.) at 1000 °C for 24 h.^40^ Bi_3_BaMNb_2_O_11_X (M = Sr, Ca, X = Cl, I) were also prepared via similar procedures, where Bi_3_MNb_2_O_9_ (M = Sr, Ca) were used as precursors. SrCO_3_ (FUJIFILM Wako Pure Chemical Corp.) or CaCO_3_ (FUJIFILM Wako Pure Chemical Corp.) was used instead of BaCO_3_. The mixture of BaBiO_2_X (X = Cl, I) and Bi_3_MNb_2_O_9_ was calcined in an evacuated silica tube at 800 °C (M = Sr) or 850 °C (M = Ca) for 20 h.

Bi_4_NbO_8_X (X = Cl, I) was synthesized by a solid-state reaction of BiNbO_7_ and BiOX (X = Cl, I). These precursors were thoroughly mixed with a molar ratio of 1:1.05 and heated in an evaluated silica tube at 700 °C for 10 h. The BiNbO_7_ precursor was prepared by calcining the mixture of Bi_2_O_3_ and Nb_2_O_5_ at 800 °C for 5 h. Bi_5_BaTi_3_O_14_I was synthesized by calcination of Bi_2_O_3_, TiO_2_ (FUJIFILM Wako Pure Chemical Corp.), and BaBiO_2_X (10 mol % excess) in an evaluated silica tube at 850 °C for 20 h. Bi_6_NbWO_14_X (X = Cl, I) was prepared by calcining the mixture of Bi_2_O_3_, Nb_2_O_5_, WO_3_ (Kojundo Chemicals), and BiOX (5 mol % excess) in an evaluated silica tube at 800 °C for 20 h.

SrTiO_3_:Rh^41^ was prepared by the solid-state reaction. A mixture of TiO_2_, SrCO_3_, and Rh_2_O_3_ (Ti/Sr/Rh = 1:1.07:0.01) was calcined in air at 800 °C for 1 h and subsequently at 1000 °C for 10 h. A Ru-based cocatalyst (0.7 wt % calculated as metal) was loaded onto SrTiO_3_:Rh by photodeposition using RuCl_3_·*n*H_2_O (FUJIFILM Wako Pure Chemical Corp.) as a precursor.^38^

### Material Modeling

All calculations were performed using density functional theory (DFT) within the periodic boundary condition using the Vienna Ab Initio Simulation Package (VASP).^42^ The projector augmented wave (PAW) method was employed. The Perdew–Burke–Ernzerhof (PBE)^43^ formulation of the generalized gradient approximation (GGA) was employed as the exchange–correlation functional with D3 dispersion. The atomic positions were optimized until the Hellman–Feynman forces on each atom were below 0.001 eV Å^–1^. The energy convergence criterion was set to 10^–5^ eV. A plane-wave energy cutoff of 600 eV was used for all calculations. The 4 × 4 × 1, 7 × 7 × 2, 6 × 6 × 1, 3 × 3 × 1 Γ-centered *k*-point meshes were employed for geometry optimization of Bi_4_NbO_8_X, Bi_3_Ba_2_Nb_2_O_11_X, Bi_5_BaTi_3_O_14_X, and Bi_6_NbWO_14_X, respectively, and were doubled for projected density of states (PDOS) and crystal orbital Hamilton population (COHP) calculations using the LOBSTER package.^44^ The conductivity effective mass tensors were calculated using AMSET.^45^ The dielectric constants were calculated by density functional perturbation theory (DFPT). Electronic band structure diagrams were generated using the sumo package.^46^ The Fröhlich polaron properties were solved using the Polaron Mobility package.^47^

### Characterizations

Powder XRD (MiniFlex II, Rigaku, X-ray source: Cu Kα), UV–visible diffuse reflectance spectroscopy (V-650, JASCO), and SEM-EDX (NVision 40, Carl Zeiss-SIINT) were used for characterization of samples. High-angle annular dark-field scanning transmission electron microscopy (HAADF-STEM) and annular bright-field scanning transmission electron microscopy (ABF-STEM) images were collected using a JEM-ARM200CF (JEOL Ltd., Tokyo, Japan) operating at an accelerating voltage of 200 kV and equipped with a cold field-emission gun and a Cs corrector to observe atomic columns. Elemental analysis was performed using a JEM-ARM200CF equipped with an energy-dispersive X-ray (EDX) spectroscopy system. Samples were prepared by grinding the material and depositing a few drops of the suspension onto a holey copper grid covered with a thin carbon film. SXRD patterns were collected at the BL02B2 beamline in SPring-8, Japan (λ = 0.419432 Å). NPD data was collected at RT using the BL09 Spica beamline at the Japan Proton Accelerator Research Complex (J-PARC). The collected X-ray and neutron diffraction data were analyzed using RIETAN-FP^48^ and Z-Rietveld.^49^

### Mott–Schottky Measurements

The sample was mixed with a small amount of water; then, the obtained paste was coated on a fluorine-doped tin oxide (FTO) conductive substrate via a squeezing method and dried in air at 60 °C. The Mott–Schottky plots were recorded using an electrochemical analyzer (PARSTAT2263, Princeton Applied Research). Electrochemical measurements were performed in a three-electrode cell using a Pt wire counter electrode, a Ag/AgCl reference electrode, and phosphate-buffered solution (0.1 M, pH = 6.0) with an amplitude of 10 mV and a frequency of 1 kHz.

### Photocatalytic Reactions

The photocatalytic reactions were performed in a gas closed-circulation system. Photocatalyst powders (0.1 g) were dispersed in an aqueous AgNO_3_ solution (8 mM, 100 mL) in a Pyrex top-window cell. The photocatalysts were irradiated with visible light (λ > 400 nm) through a cutoff filter (HOYA; L42) from a 300 W Xe-arc lamp (PerkinElmer; Cermax PE300BF). The quantity of the evolved gases was determined using an online gas chromatograph (thermal conductivity detector; molecular sieve 5 Å column packing; Ar carrier gas). The apparent quantum efficiency (AQE) was evaluated using a 405 nm monochromatic light-emitting diode (LED) light source (ASAHI SPECTRA, CL-1501).

For water oxidation reaction in the presence of an Fe^3+^ electron acceptor, ruthenium oxide (RuO_2_) was loaded as a cocatalyst. A small amount of sample was mixed with an aqueous solution containing Ru(III) acetylacetonate (Ru(acac)_3_) (Sigma-Aldrich) followed by heating under an Ar flow at 450 °C for 30 min.

The Z-scheme water-splitting reaction was conducted using RuO_2_-loaded Bi_6_NbWO_14_I (0.05 g) and Ru-loaded SrTiO_3_:Rh (0.05 g) as H_2_- and O_2_-evolving photocatalysts, respectively. They were suspended in an aqueous Fe(NO_3_)_3_ solution (2 mM, 100 mL). The solution pH was adjusted to ∼2.4 with diluted aqueous HNO_3_ solution. The suspension was irradiated with visible light (λ > 400 nm).

### Complexometric Titration of Fe^2+^

The amount of Fe^2+^ produced from Fe^3+^ reduction during the O_2_ evolution reaction was quantified as follows. After the photocatalytic reactions, the powdery sample was removed from the solution by filtration. The concentrations of Fe cations remaining in the solutions were determined from the absorption spectra of the solutions measured by UV–vis spectroscopy (Shimadzu, UV-1800). The reaction solution (50 μL), 2 M acetate buffer solution (2.1 mL), and 9.6 × 10^–4^ M TPTZ solution (0.7 mL) were mixed, and then the produced amount of Fe^2+^ was determined on the basis of the absorbance at 596.5 nm.

## Supporting Information Available

XRD patterns, HAADF-STEM images, SEM images, Mott–Schottky plots, DFT results, and COHP analysis data (PDF)

## Accession Codes

CCDC 2266014 contains the supplementary crystallographic data for this paper. These data can be obtained free of charge via www.ccdc.cam.ac.uk/data_request/cif, or by emailing data_request@ccdc.cam.ac.uk, or by contacting The Cambridge Crystallographic Data Centre, 12 Union Road, Cambridge CB2 1EZ, U.K.; fax: +44 1223 336033.
