# Supplementary material for: Orbital Engineering in Sillén–Aurivillius Phase Bismuth Oxyiodide Photocatalysts through Interlayer Interactions
Source: Chem Mater. 2023 Jul 12;35(14):5532–40. doi: 10.1021/acs.chemmater.3c00932 (PMC10373439; doi:10.1021/acs.chemmater.3c00932)
Supplement: Supplementary file 1 — cm3c00932_si_001.pdf [file cm3c00932_si_001.pdf]

Supporting Information

Orbital Engineering in Sillén-Aurivillius Phase Bismuth Oxyiodide Photocatalysts through Interlayer Interactions

Kanta Ogawa,[1] Hajime Suzuki,[2] Aron Walsh[1,*] and Ryu Abe[2,*]

[1] Centre for Processable Electronics and Department of Materials, Imperial College London, Exhibition Road, London SW7 2AZ, UK

[2] Department of Energy and Hydrocarbon Chemistry, Graduate School of Engineering, Kyoto University, Nishikyo-ku, Kyoto 615-8510, Japan

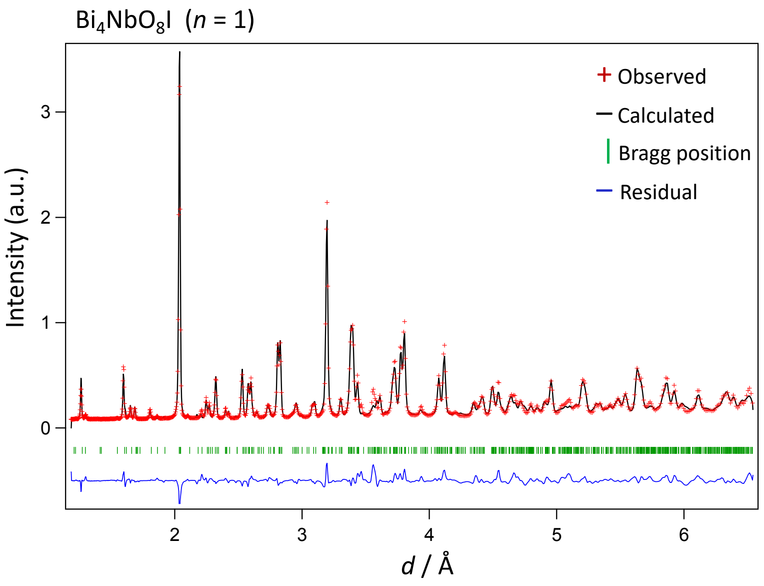

Figure S1. Rietveld analysis of Bi_4NbO_8I using the neutron power diffraction pattern collected at room temperature. The reliability factors are Rp = 6.43%, Rwp = 9.10%.

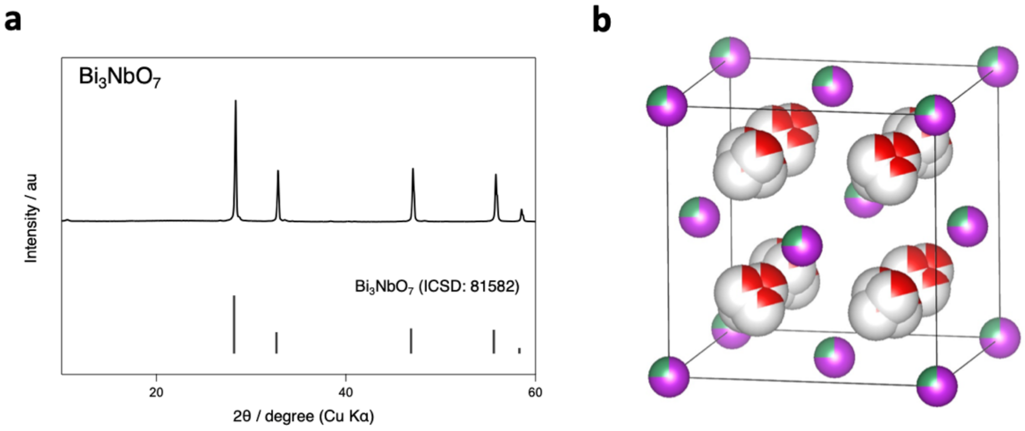

Figure S2. (a) XRD pattern and (b) crystal structure of Bi_3NbO_7, which is a precursor for the synthesis of Bi_4NbO_8I.

Table S1. Structural parameters of Bi_4NbO_8I.[a]

Atom	Wyckoff position	*x*	*y*	*z*	B_{iso} (Å^2)
Nb1	4a	-0.01173(17)	0.2496(2)	0.25064(11)	0.329(13)
Bi1	4a	0.0200(4)	0.7681(2)	0.16379(5)	3.73(5)
Bi2	4a	0.0022(3)	0.24976(18)	0.42332(7)	0.19(3)
Bi3	4a	0.0127(3)	0.80127(18)	0.33819(4)	1.06(3)
Bi4	4a	0.0083(3)	0.2479(2)	0.07777(7)	1.00(4)
I1	4a	0.5161(3)	0.2471(3)	-0.00348(9)	0.90(2)
O1	4a	0.7712(3)	0.5102(4)	0.38743(7)	0.60(5)
O2	4a	0.2498(3)	0.4930(3)	0.38566(6)	0.13(4)
O3	4a	0.7378(4)	-0.0188(4)	0.39082(5)	0.04(4)
O4	4a	0.2480(4)	0.0360(4)	0.38754(6)	0.38(4)
O5	4a	0.4105(3)	0.8085(3)	0.31313(8)	1.71(5)
O6	4a	0.4182(3)	0.6687(3)	0.18540(8)	1.00(4)
O7	4a	0.2338(5)	-0.0145(4)	0.24467(10)	8.59(10)
O8	4a	-0.2556(3)	-0.0290(3)	0.23513(7)	2.32(5)

[a]Space group: $P2_1cn$; a = 5.53249(2)Å, b = 5.59200(2) Å, c = 29.8029(1) Å

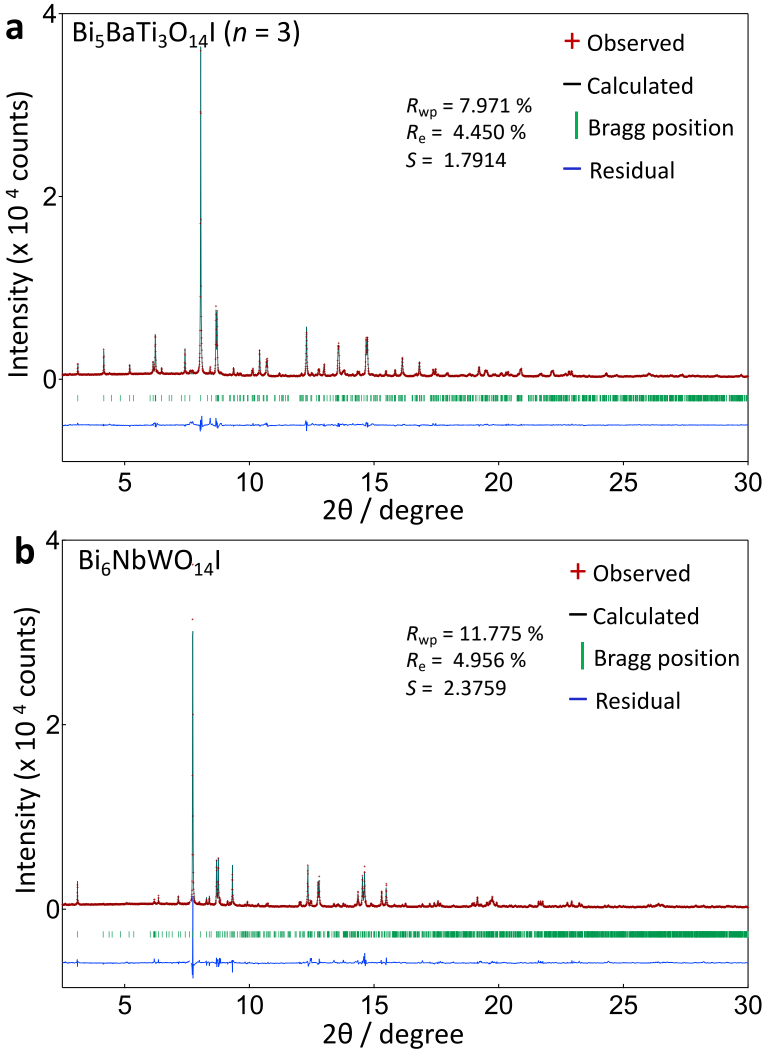

Figure S3. Rietveld refinement of SXRD pattern of (a) $Bi_5BaTi_3O_{14}I$ and (b) $Bi_6NbWO_{14}I$. The positions of oxygens were not refined. For $Bi_6NbWO_{14}I$, an impurity peak around 2θ = 7.6 was excluded for the refinement.

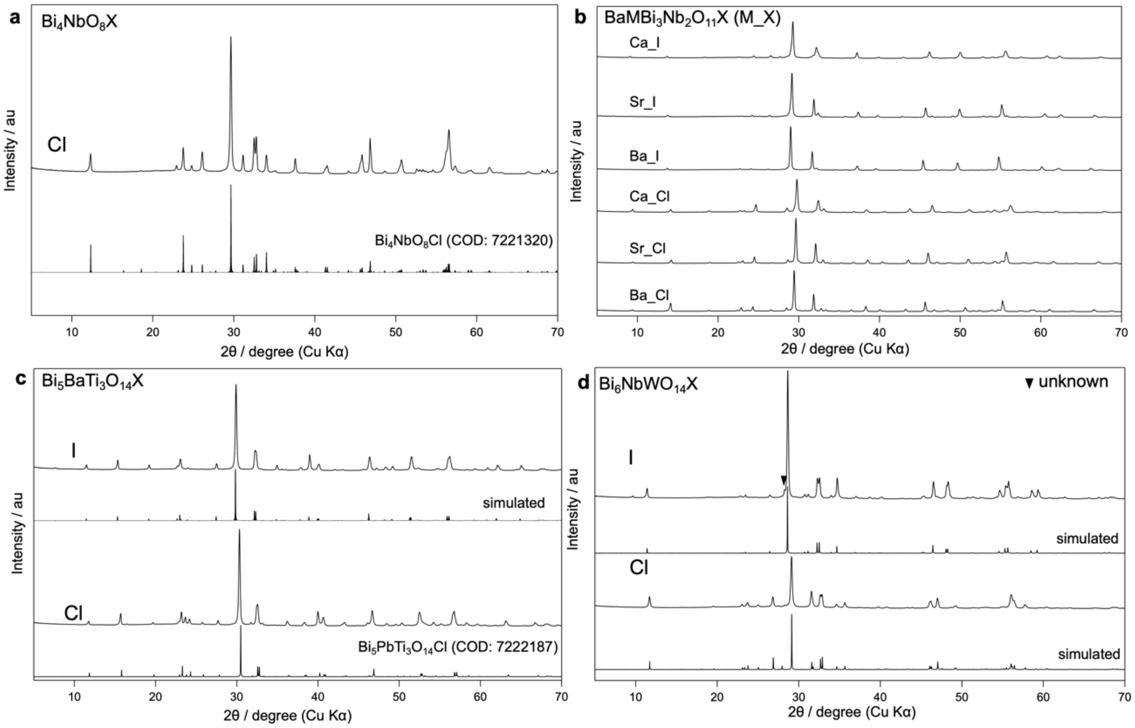

Figure S4. XRD patterns of (a) Bi_4NbO_8Cl, (b) $BaMBi_3Nb_2O_{11}X$ (M = Ca, Sr, Ba; X = Cl, I), (c) $Bi_5BaTi_3O_{14}Cl$, and (d) $Bi_6NbWO_{14}X$. Diffraction patterns of $Bi_5BaTi_3O_{14}I$ and $Bi_6NbWO_{14}X$ were simulated by using VESTA[1] with space groups according to literature[2,3] (*P2an* and *Pca2₁,* respectively) and lattice parameters determined by Le Bail refinement as comparisons.

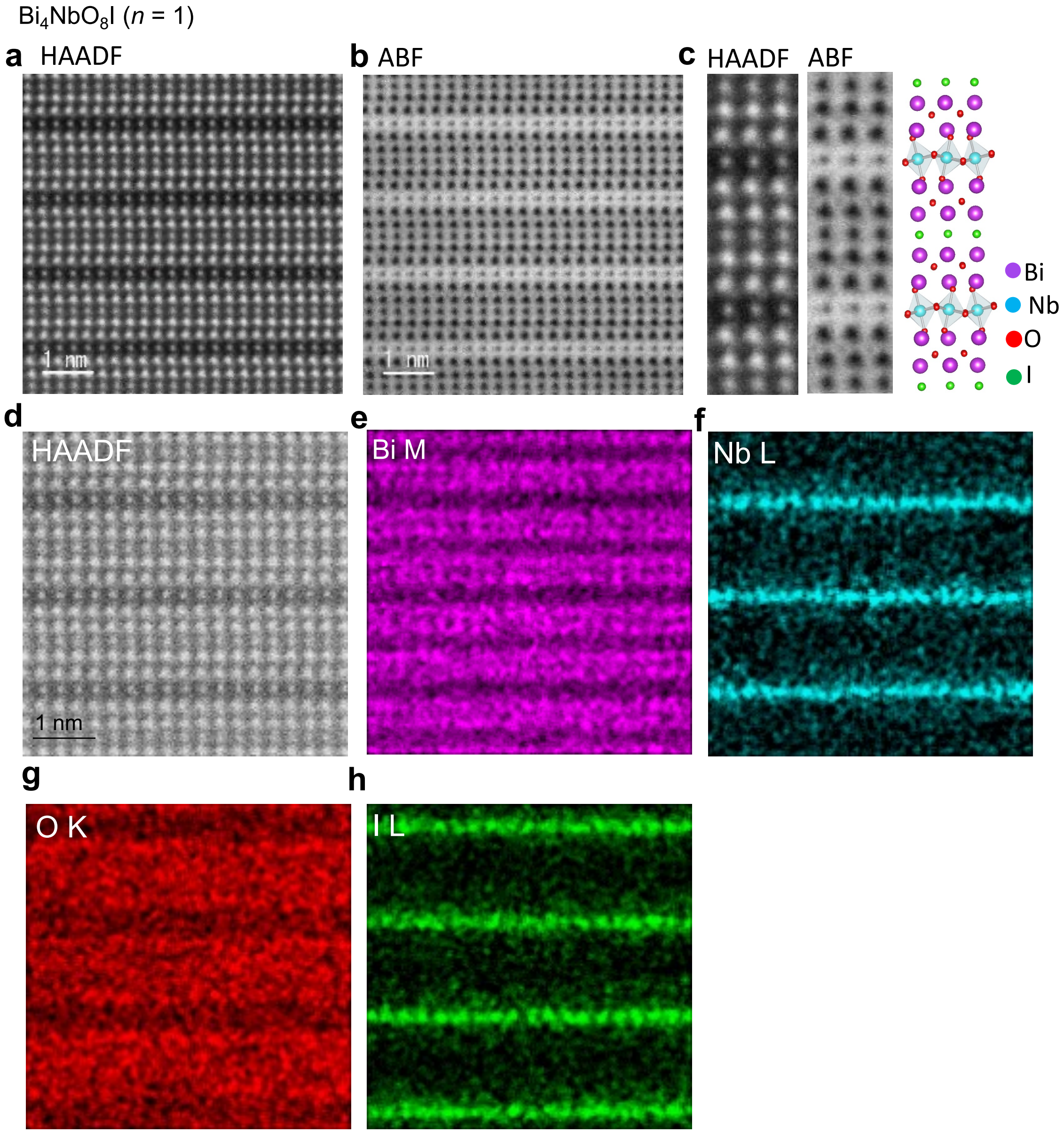

Figure S5. (a) HAADF and (b) ABF STEM images of Bi_4NbO_8I along the $[100]_t$ direction. (c) Zoomed view of the STEM image, along with the corresponding Bi_4NbO_8I crystal structure. (d-h) STEM-EDX atomic resolution elemental maps for (e) Bi, (f) Nb, (g) O, (h) I.

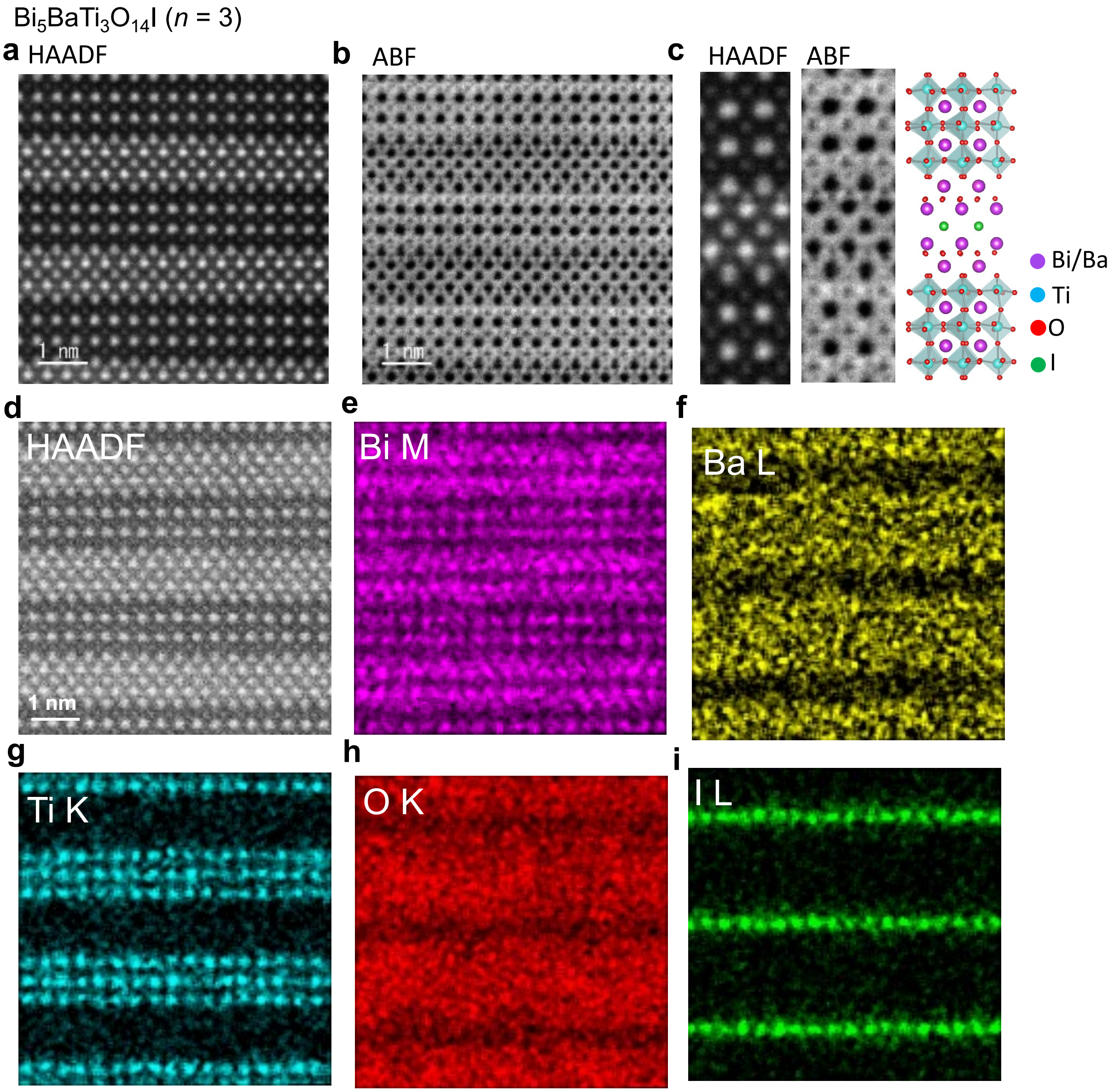

Figure S6. (a) HAADF and (b) ABF STEM images of $Bi_5BaTi_3O_{14}I$ along the $[110]_t$ direction. (c) Zoomed view of the STEM image, along with the corresponding $Bi_5BaTi_3O_{14}I$ crystal structure. (d-i) STEM-EDX atomic resolution elemental maps for (e) Bi, (f) Ba, (g) Ti, (h) O, (i) I.

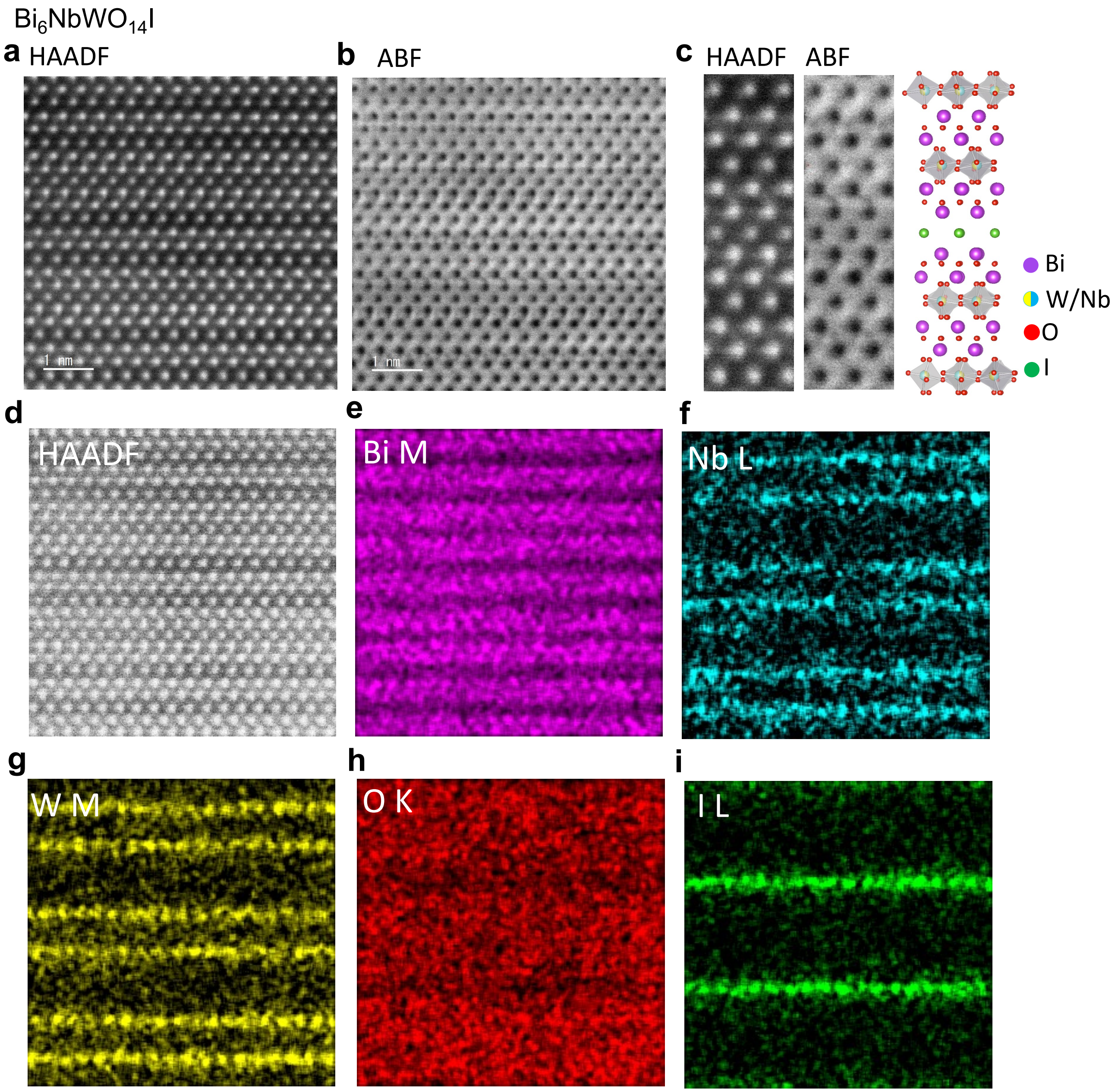

Figure S7. (a) HAADF and (b) ABF STEM images of $Bi_6NbWO_{14}I$ along the $[110]_t$ direction. (c) Zoomed view of the STEM image, along with the corresponding $Bi_6NbWO_{14}I$ crystal structure. (d-i) STEM-EDX atomic resolution elemental maps for (e) Bi, (f) Nb, (g) W, (h) O, (i) I.

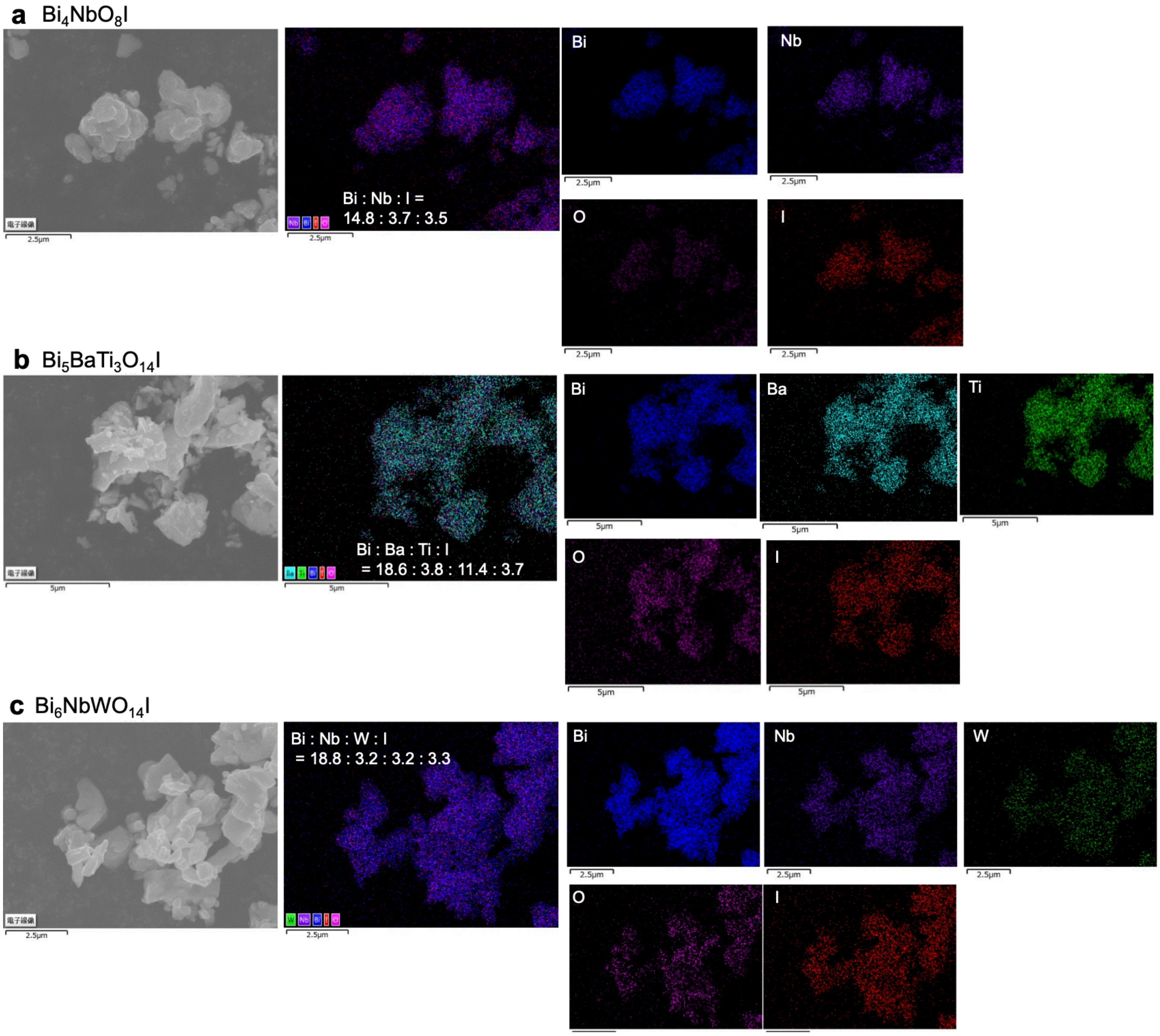

Figure S8. SEM images and SEM-EDS elemental mapping of (a) Bi_4NbO_8I, (b) $Bi_5BaTi_3O_{14}I$, and (c) $Bi_6NbWO_{14}I$ along with elemental molar ratios determined SEM-EDS analysis.

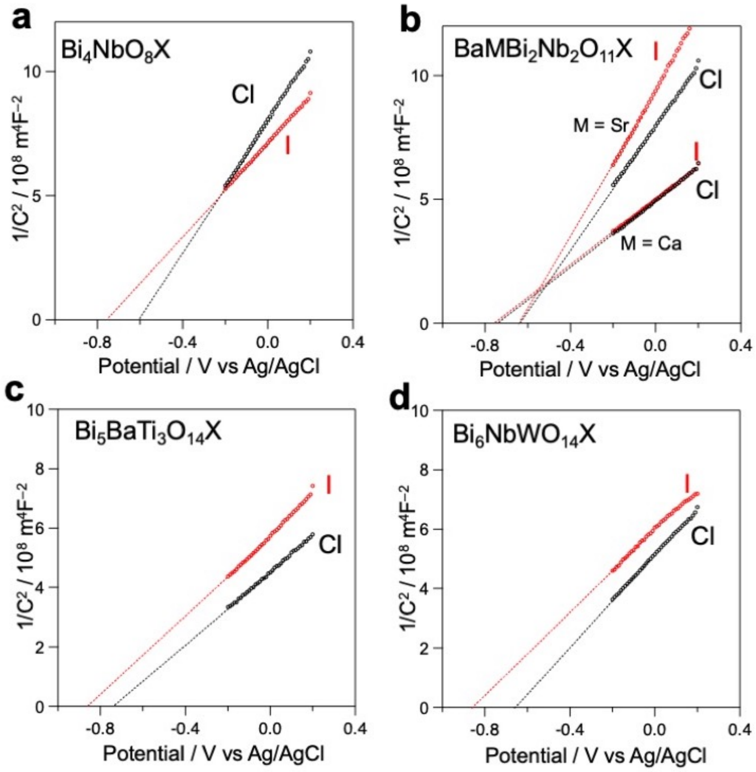

Figure S9. Mott–Schottky plots for (a) Bi_4NbO_8X, (b) $BaMBi_3Nb_2O_{11}X$ (M = Ba, Sr, Ca), (c) $Bi_5BaTi_3O_{14}X$, and (d) $Bi_6NbWO_{14}X$.

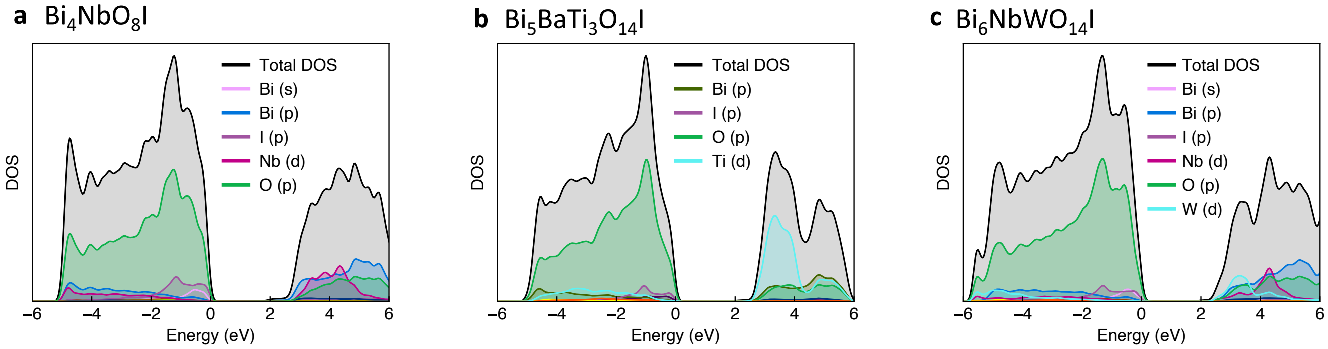

Figure S10. Electronic density of states projected onto atomic orbitals for (a) Bi_4NbO_8I, (b) $Bi_5BaTi_3O_{14}I$ and (c) $Bi_6NbWO_{14}I$.

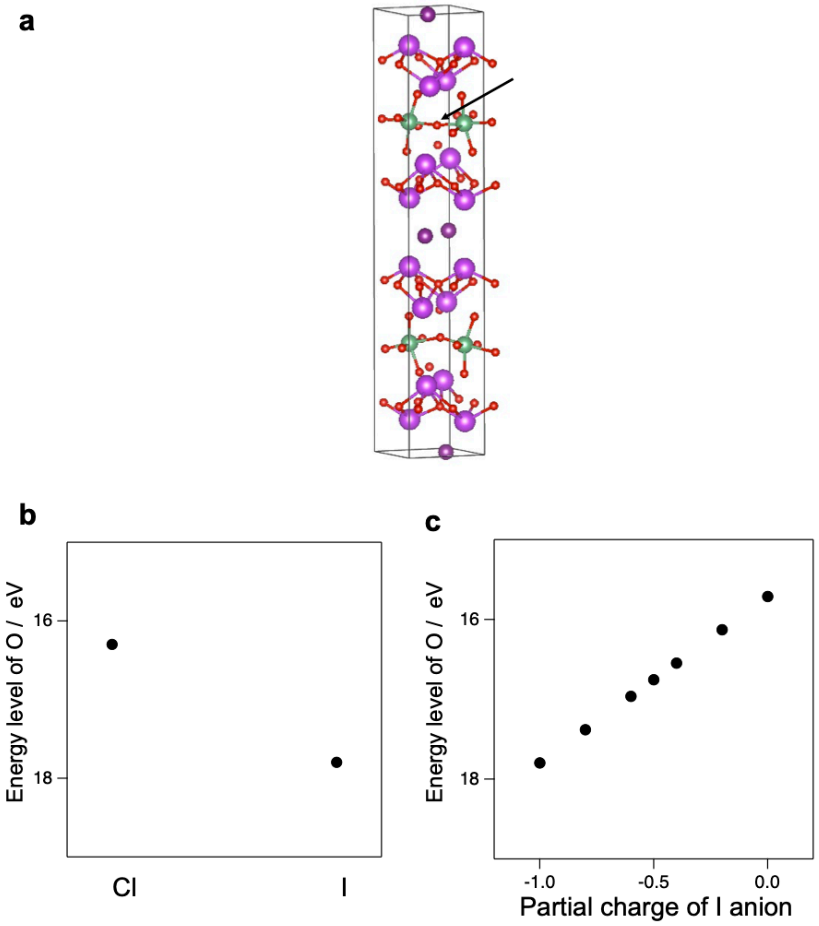

Figure S11. (b) Ionic orbital energy levels of O^{2-} at an O site in Bi_4NbO_8I (a) calculated by the sum of Madelung potential and second electron affinity of oxygen. (c) The effect of the partial charge of I anion on the energy levels of O1 site in the Madelung potential analysis.

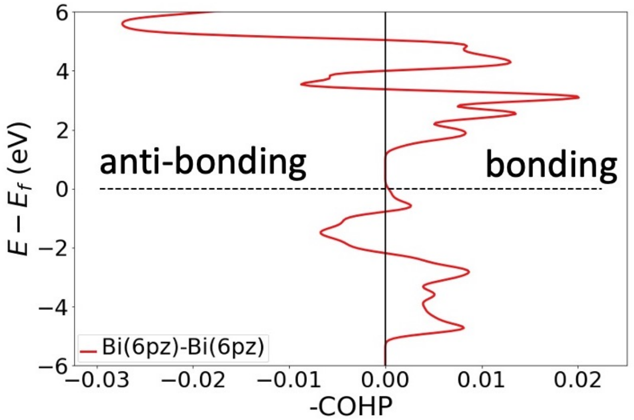

Figure S12. Crystal orbital Hamilton populations (COHP) for interlayer Bi(6pz)–Bi(6pz) interaction in Bi_4NbO_8Cl.

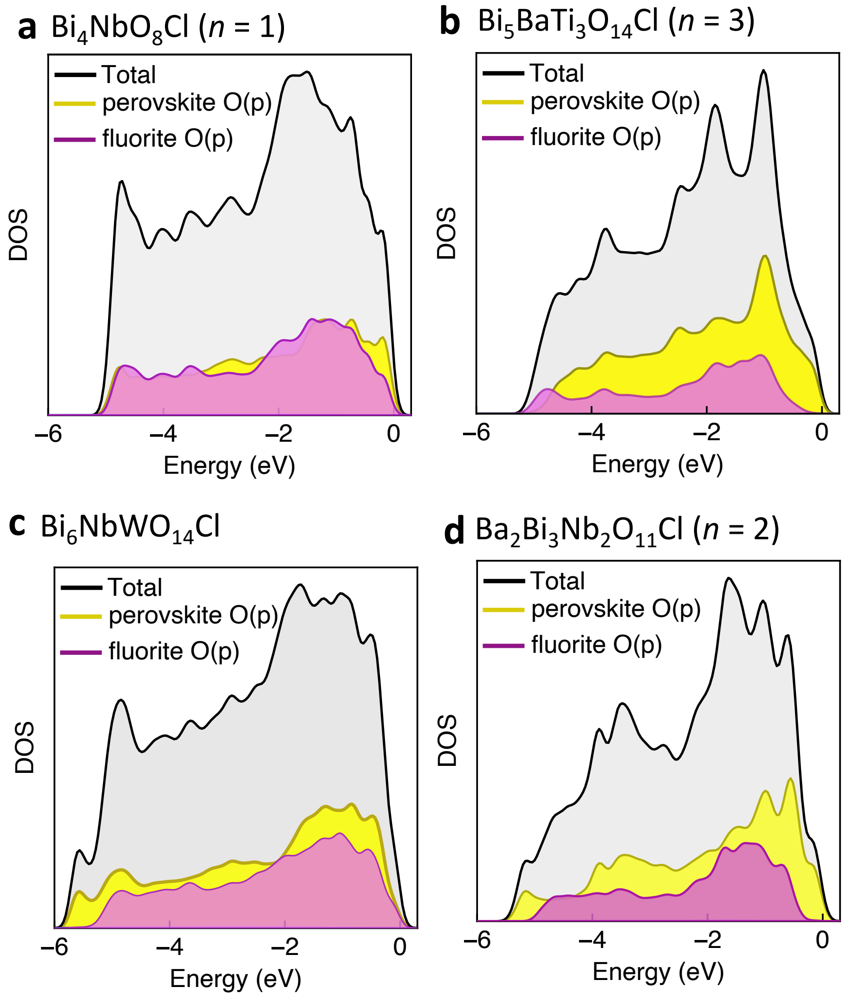

Figure S13. PDOS for the oxygens in the perovskite layer (yellow) and the fluorite layer (purple) of (a) Bi_4NbO_8Cl (n = 1), (b) $Bi_5BaTi_3O_{14}Cl$ (n = 3), and (c) $Bi_6NbWO_{14}Cl$, and (d) $Ba_2Bi_3Nb_2O_{11}Cl$ (n = 2).

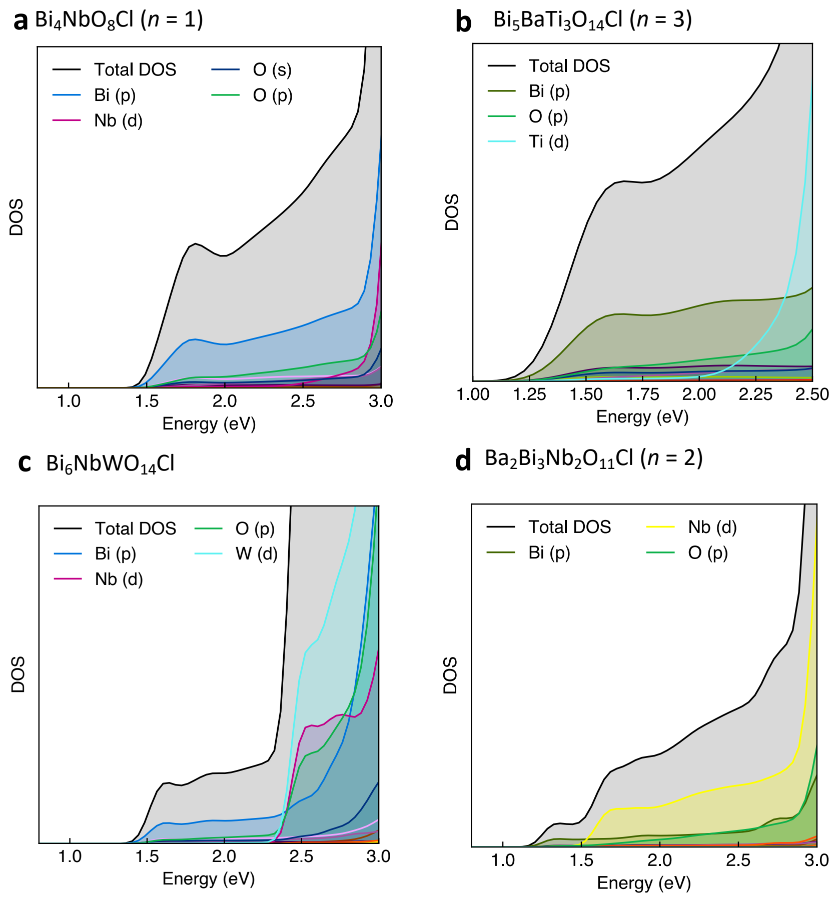

Figure S14. PDOS for the CB of (a) Bi_4NbO_8Cl (n = 1), (b) $Bi_5BaTi_3O_{14}Cl$ (n = 3), and (c) $Bi_6NbWO_{14}Cl$, and (d) $Ba_2Bi_3Nb_2O_{11}Cl$ (n = 2).

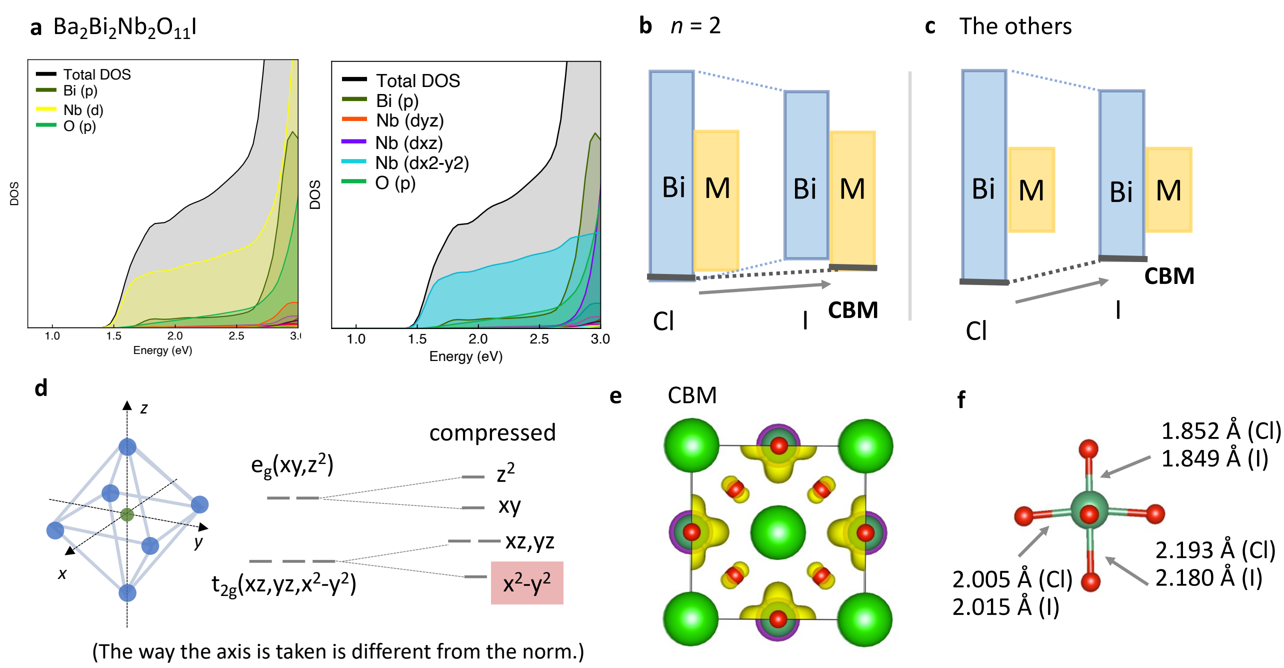

Figure S15. (a) PDOS for the CB of $Ba_2Bi_3Nb_2O_{11}I$ (n = 2). (b, c) The composition change of the CBM of n = 2 via iodine introduction and resultant narrowed Bi-6p band width. (d) Coordination environment of Nb in perovskite layer of n = 2. (e) Charge distribution of the lower part of the CBM for $Ba_2Bi_3Nb_2O_{11}I$ (n = 2). (f) Bond length of the Nb-O in $Ba_2Bi_3Nb_2O_{11}X$ from DFT calculation.

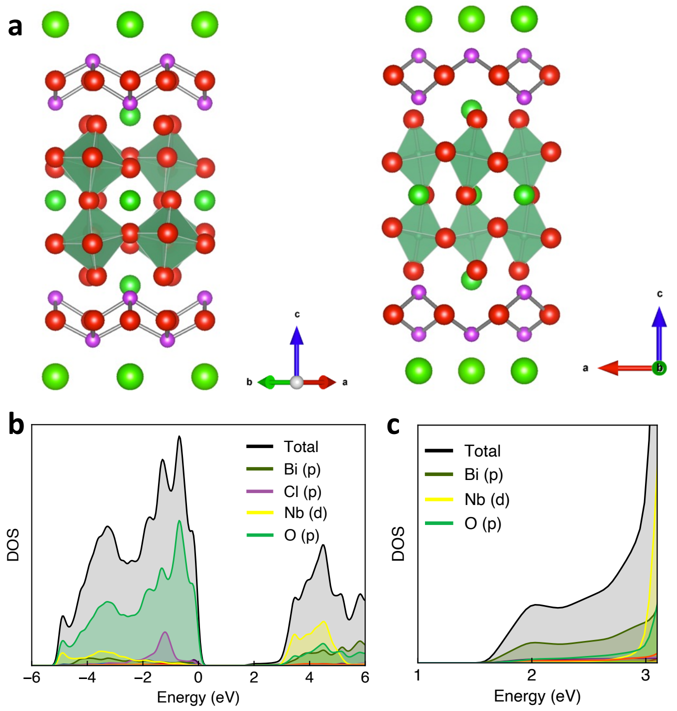

Figure S16. (a) Crystal Structure and (b, c) PDOS of $Bi_3Ba_2Nb_2O_{11}X$ (n = 2) with distortion in the perovskite layer.

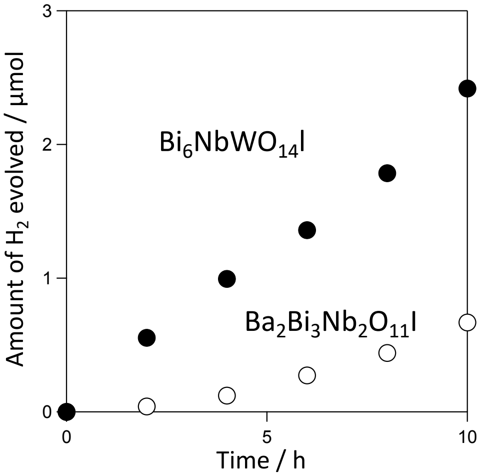

Figure S17. Time courses of H_2 evolution over Rh (1 wt%)-loaded $Ba_2Bi_3Nb_2O_{11}I$ and $Bi_6NbWO_{14}I$ in methanol-water mixed solution (1:4, v/v) under UV-visible light irradiation (λ > 300 nm). Rh cocatalyst was loaded on the photocatalysts by photoreduction method using $Rh(NO_3)_3$ as the metal source and methanol as a hole scavenger.

Table S2. Apparent quantum efficiencies (AQE) at 405 nm for water oxidation over the layered perovskite oxyiodides.

no.	compound	AQE	
		I	Cl
1	Bi_4NbO_8X (n = 1)	1.18	0.37
2	$Bi_5BaTi_3O_{14}X$ (n = 3)	0.68	0.37
3	$Bi_6NbWO_{14}X$	1.54	1.29

Table S3. Calculated effective mass of the layered perovskite oxyiodides. The dispersion along *z* directions is too flat to determine an effective mass.

material			x	y
Bi_4NbO_8X (n = 1)	X = Cl	m_e^*/m_0	0.22	0.21
		m_h^*/m_0	2.87	2.39
	I	m_e^*/m_0	0.19	0.19
		m_h^*/m_0	0.57	0.76
$Ba_2Bi_3Nb_2O_{11}X$ (n = 2)	X = Cl	m_e^*/m_0	0.21	
		m_h^*/m_0	2.09	
	I	m_e^*/m_0	0.29	
		m_h^*/m_0	0.81	
$Bi_5BaTi_3O_{14}X$ (n = 3)	X = Cl	m_e^*/m_0	0.18	0.19
		m_h^*/m_0	2.42	2.41
	I	m_e^*/m_0	0.17	0.17
		m_h^*/m_0	2.1	2.1
$Bi_6NbWO_{14}X$	X = Cl	m_e^*/m_0	0.25	0.25
		m_h^*/m_0	4.63	4.80
	I	m_e^*/m_0	0.17	0.19
		m_h^*/m_0	2.76	2.39

Table S4. Calculated dielectric constant of the layered perovskite oxyiodides, where ε_{ion} represents ionic contribution and ε_{∞} represents electronic contribution.

material			x	y
Bi_4NbO_8X (n = 1)	X = Cl	ε_{ion}	35.98	35.98
		ε_{∞}	6.42	6.18
	I	ε_{ion}	35.93	30.60
		ε_{∞}	7.06	6.79
$Ba_2Bi_3Nb_2O_{11}X$ (n =2)	X = Cl	ε_{ion}	99.18	
		ε_{∞}	5.98	
	I	ε_{ion}	211.46	
		ε_{∞}	6.51	
$Bi_5BaTi_3O_{14}X$ (n = 3)	X = Cl	ε_{ion}	67.37	68.29
		ε_{∞}	6.50	6.51
	I	ε_{ion}	64.72	65.63
		ε_{∞}	6.84	6.79
$Bi_6NbWO_{14}X$	X = Cl	ε_{ion}	26.27	35.25
		ε_{∞}	6.21	6.42
	I	ε_{ion}	21.64	30.85
		ε_{∞}	6.21	6.42

Supporting references

1 K. Momma and F. Izumi, *J Appl Crystallogr*, 2011, **44**, 1272–1276.

2 D. Ozaki, H. Suzuki, A. Nakada, M. Higashi, O. Tomita, H. Kageyama and R. Abe, *Chem. Lett.*, 2020, **49**, 978–981.

3 H. Kunioku, M. Higashi, C. Tassel, D. Kato, O. Tomita, H. Kageyama and R. Abe, *Chem. Lett.*, 2017, **46**, 583–586.
